# Exposure of Metal Oxide Nanoparticles on the Bioluminescence Process of *P_u_-* and *P_m_-lux* Recombinant *P. putida mt-2* Strains

**DOI:** 10.3390/nano11112822

**Published:** 2021-10-24

**Authors:** In Chul Kong, Kyung-Seok Ko, Sohyeon Lee, Dong-Chan Koh, Robert Burlage

**Affiliations:** 1 Department of Environmental Engineering, Yeungnam University, Gyungsan 38541, Korea; ickong@ynu.ac.kr (I.C.K.); lswsh0803@naver.com (S.L.); 2 Groundwater Department, Geologic Environment Division, Korea Institute of Geoscience and Mineral Resources (KIGAM), Daejeon 34132, Korea; chankoh@kigam.re.kr; 3 Department of Pharmaceutical and Administrative Sciences, Concordia University, Mequon, WI 53097, USA; Robert.burlage@cuw.edu

**Keywords:** bioluminescence, inducer, nanoparticles, *P_m_-* and *P_u_-lux*, recombinant

## Abstract

Comparison of the effects of metal oxide nanoparticles (NPs; CuO, NiO, ZnO, TiO_2_, and Al_2_O_3_) on different bioluminescence processes was evaluated using two recombinant (*P_m_-lux* and *P_u_-lux*) strains of *Pseudomonas putida mt-2* with same inducer exposure. Different sensitivities and responses were observed according to the type of NPs and recombinant strains. EC_50_ values were determined. The negative effects on the bioluminescence activity of the *P_m_-lux* strain was greater than for the *P_u_-lux* strains for all NPs tested. EC_50_ values for the *P_m_-lux* strain were 1.7- to 6.2-fold lower (corresponding to high inhibition) than for *P_u_-lux*. ZnO NP caused the greatest inhibition among the tested NPs in both strains, showing approximately 11 times less EC_50s_ of CuO, which appeared as the least inhibited. Although NPs showed different sensitivities depending on the bioluminescence process, similar orders of EC_50s_ for both strains were observed as follows: ZnO > NiO, Al_2_O_3_ > TiO_2_ > CuO. More detailed in-depth systematic approaches, including in the field of molecular mechanisms, is needed to evaluate the accurate effect mechanisms involved in both bioluminescence metabolic processes.

## 1. Introduction

Petroleum-based hydrocarbons are an important source of energy and are also used as raw materials for the production of various organic compounds, but they are becoming some of the most important and common pollutants in ecosystems [1,2,3]. Although a large number of remediation technologies are generally applied to clean up such contaminated sites, biomonitoring may be an effective remediation strategy in some cases, especially for groundwater and subsurface contamination where contaminant plumes are retained within sites [4]. This requires a variety of biomonitoring technologies including bioassays and biosensors, which provide information about the impact of contaminants on organisms and ecosystems [5,6]. Various microorganisms and higher life (i.e., fishes, plants, and invertebrates) are used for biomonitoring, while microbial processes are especially attractive for contaminated sites [7,8]. Among various microbial processes, methods using the activity of whole cell, specific enzymes, and intact or recombinant gene expression have been adopted for assessing the environmental pollutants by regulatory and monitoring agencies because they are simple, rapid, and low cost. These also can be used for preliminary assessment and proper complementary approaches for interpretation of the chemical and physical analysis [4,9]. Among the applicable microbial activities, recombinated genes and their regulation have resulted in interest in tools for the development of environmental monitoring. The *lux* genes found in *Vibrio fischeri* (*lux*CDABE) (recently named as *Aliivibrio fischeri*) is one of the most applicable reporter genes [4]. Recombinant strains containing *lux* genes can be used for real-time, non-destructive assays based on bioluminescence (visible light) production. Bacteria containing recombinant *lux*-genes and the TOL plasmid, that encodes enzymes converting toluene analogs (notably gasoline components) into non-toxic intermediates, have been shown to be effective bioindicators of contaminants [10,11]. Bioluminescence is related to bacterial metabolic activity, and could be a sensitive indicator of bacterial metabolic status [4]. When these *lux* genes combined with other genes that are activated in the presence of specific contaminants, the *lux* gene sequence of *V. fischeri* acts as an effective bioreporter for the applications of environmental biomonitoring. One example of bioluminescence induction is *P. putida* mt-2 KG1206 and RB 1401, which contain the *P_m_-lux* and *P_u_*-*lux* recombinant plasmid with TOL plasmid, respectively. Two regulatory genes, XylS and XylR control the catabolic activity of the TOL plasmid genes, and these two genes act as transcriptional activators on two operons of the upper and lower pathways [10,11]. 

The two strains used for this investigation can produce bioluminescence in the presence of toluene analogs and their intermediates, and therefore, can be used for preliminary biomonitoring approaches of the gasoline-contaminated sites. Valuable information about the bioavailability of contaminants in the environment can be generated by these bioluminescence activities. These results can serve as useful alternatives compared to expensive analytical methods for *on-site* analysis or in situ monitoring. 

Biomonitoring studies have generally focused on individual contaminants under laboratory conditions. However, the contaminated site is generally exposed to a mixture of pollutants rather than one. Therefore, analyzing mixtures will portray the realistic conditions of biomonitoring sites. The commercial products of NPs are increasingly found in the environment due to the fast growing nanotechnology field, a consequence of their global economic importance. They are one of the possible co-pollutants with hydrocarbons [12,13,14]. NPs technologies have progressed due to their unique and distinctive characteristics (high specific surface area, small size distribution, high dispensability, etc.) and are entering many industrial areas such as electronics, energy, medicine, plastics, and aerospace [15]. The promise of any new technology, however, must be weighed against the possibility of generating exceptional hazards for the environment [16,17]. Therefore, evaluating the possible effects of NPs on the organisms used for the biomonitoring of specific contaminants in the contaminated site is needed. Among the various groups of NPs, a wide range of metal-based varieties are common in industry [18]. The effect of NPs on organisms is closely related to their unique characteristics of nano-scale, high surface area, modified surface, and radical formation, especially when considering aggregation of insoluble particles and soluble metals, which greatly affect the state of metal NPs in solution. These characteristics may impact the bioavailability, uptake, and toxicity of metal-based NPs [19]. 

In this research, comparisons of the effects of NPs were investigated based on the bioluminescence activity of two different recombinant strains, which can be used as bioreporters for the biomonitoring sites. Two different recombinant strains of *P. putida* mt-2, called KG1206 (*P_m_-lux*) and RB1401 (*P_u_-lux*), in which expression of the *lux* gene was placed under the control of a TOL plasmid promoter (*P_m_* or *P_u_*), were adopted for investigation. NPs are possible pollutants along with petroleum hydrocarbons, creating a challenging environment for bioreporter analysis.

## 2. Materials and Methods

### 2.1. Characteristics of Recombinant Strains 

**Figure 1:**
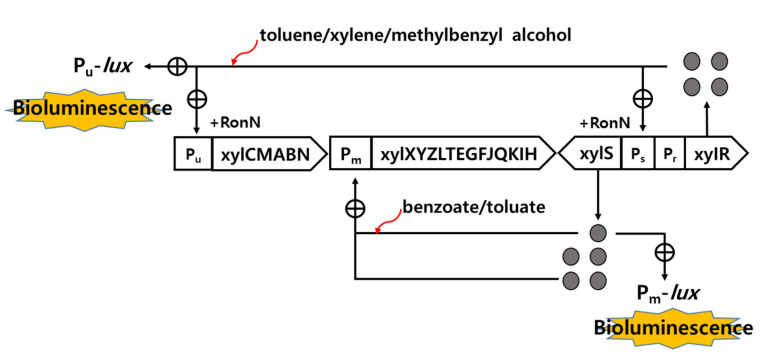
Regulation of recombinant *P_u_-lux* and *P_m_-lux* genes (⊕: positive control; ●: regulatory protein) (modified from [9]).

The recombinant strains *Pseudomonas putida* mt-2 RB1401 and KG1206 contain the intact TOL plasmid and *P_u_-lux* and *P_m_-lux* fused plasmid, where *P_u_* and *P_m_* are the promoters of the upper and lower operon of the pWW0 (TOL plasmid), respectively. These are responsible for bioluminescence production in the presence of toluene analogs and their intermediates [9,20]. Details of these *lux* recombinants are shown in Figure 1. Toluene and xylene isomers activate XylR regulatory protein, whereas their intermediates (benzoate and *m*-toluate), activate XylS regulatory protein. Both inducer-activated XylR and XylS proteins positively control their promoters P_u_ and P_m_, respectively. XylR controls P_u_ and, also, induces expression of XylS gene, which is responsible for the production of XylS protein as well as regulation of P_m_. For the KG1206 strain, inducer-activated XylS protein enhances interactions with the promoter P_m_ to produce bioluminescence from the *P_m_*-*lux* gene fusion in the recombinant strain [9]. 

### 2.2. Culture Conditions and Bioluminescence Activity 

Tested strains were kept at −70 °C using standard procedures [21]. Strains were grown overnight in Luria-Bertani (LB) medium at 27 °C with shaking (130 rpm). A 1:50 dilution in LB medium was allowed to grow until the optical density was approximately (OD_600_) 0.6 [9]. An equal mixture of minimum salt medium and a log phase culture grown in LB was distributed in serum vials (9.9 mL) with inducer (0.1 mL). Vials were sealed with septa to prevent loss of the volatile organic compounds (inducers) [11]. In this experiment, *o*-chlorotoluene (CT) was used as an inducer with final exposure concentrations of 0.1 to 10 mM. During the incubation periods (generally 5 h), bioluminescence production was measured every 30 min using a Turner 20/20 Luminometer (Sunnyvale, CA, USA), where the maximum detection limit was 9999 RLU (relative light units). 

### 2.3. Effects of NPs on Bioluminescence Activity and Metal Analysis

Five tested metal oxide NPs showed following characteristics of size, density, and surface area: CuO (30–50 nm, 6.40 g/cm^3^, 13.1 m^2^/g) and NiO (30 nm, 6.67 g/cm^3^, 50–100 m^2^/g) were obtained from Nanostructured and Amorphous Materials (Houston, TX, USA); ZnO (40–100 nm, 5.61 g/cm^3^, 10–25 m^2^/g), TiO_2_ (<25 nm, 3.95 g/cm^3^, 75–85 m^2^/g), and Al_2_O_3_ (40–50 nm, 3.965 g/cm^3^, 32–40 m^2^/g) were obtained from Alfa Aesar (Tewksbury, MA, USA). Directly suspended NPs in purified water (pH 7.8) were dispersed for approximately 10 min (40 Hz) by ultrasonic vibration (DH.D250H, DAIHAN, Korea) prior to use. To determine the effects of NPs, the serum vials, which contained culture (0.8 mL) and inducer (0.1 mL *o*-CT, final concentration 1 mM), were amended with an appropriate concentration of 0.1 mL NP. Concentrations tested for each NP were determined based on a preliminary test as shown in Table S1. The culture was incubated by shaking (130 rpm) at 27 °C after adding various concentrations of NPs. During the incubation periods (generally 3 h), bioluminescence production was measured in triplicate every 0.5 h. The EC_50_ (effective chemical concentration at which 50% of its effect is observed) values of NPs on bioluminescence activity were estimated using the program SPEARMAN, which is distributed by the US EPA. At the end of the incubation, the solution samples of some experimental sets were filtered (0.45 µm) to measure the concentration of dissolved metal ions using an inductively coupled plasma optical emission spectrometer (Optima 7300DV; Perkin-Elmer Inc., Shelton, CT, USA).

## 3. Results and Discussion

Previously we reported the bioluminescence activities of two recombinant strains (KG1206 and RB1401) in the presence of toluene, xylene isomers, methylbenzyl alcohol, and their metabolic intermediates (benzoate, *m*-toluate) under various conditions [9,22]. In this investigation, we compare the effects of NPs on the bioluminescence processes of these two different recombinant strains, which were controlled by different regulatory proteins, while they are induced with *o*-CT. This inducer had not been examined in our previous investigation, but like toluene, it directly activates the XylR regulatory protein, which controls the P_u_ promoter positively, resulting in the bioluminescence production from the *P_u_-lux* recombinant gene of the RB1401 strain. This activated XylR which also induces expression from the P_s_ (XylS promoter) and indirectly activates the XylS regulatory protein, resulting in the production of bioluminescence from the *P_m_-lux* fusion gene of the KG1206 strain [9,20,23]. In addition, a catabolic intermediate of *o*-CT may also directly activate the XylS regulatory protein, resulting in the production of bioluminescence from the *P_m_-lux* gene of KG1206. A separate report described the transcription of the P_m_ promoter when XylS is induced by a sufficient concentration of XylR in the presence of direct inducer [24]. 

### 3.1. Bioluminescence Activity of P_m_- and P_u_-lux Gene Fusion to Inducer, o-CT 

**Figure 2:**
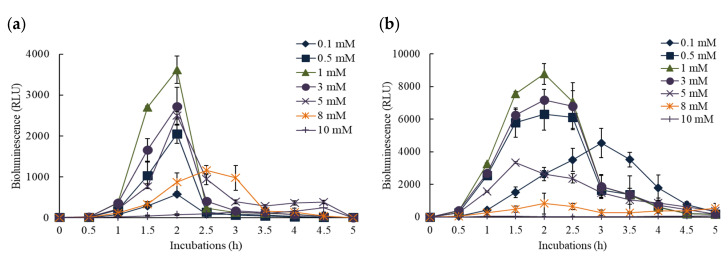
The time course of bioluminescence activity with different initial concentration of *o*-CT. (**a**) KG1206 0.1 mM–5 mM; (**b**) RB1401 0.1 mM–10 mM.

To examine the changes of bioluminescence activity in KG1206 and RB1401 with time course, cultures were exposed to various concentrations of *o*-CT ranging from 0 to 10 mM over a period of 5 h (Figure 2). No observable bioluminescence (less than 10 RLU during the exposure period) activity was detected throughout the 5 h of incubation in negative controls. The maximum bioluminescence activity was observed after 2 h incubation for both KG1206 and RB1401, followed by a sharp decrease of bioluminescence activity, and generally bioluminescence activity lasted for approximately 3–4 h. All these activities differ slightly on initial inducer concentration, as was previously published [9]. For example, when KG1206 was exposed to 3 mM of *o*-CT the maximum bioluminescence was observed at 2 h after induction (2725 ± 461 RLU). For RB1401 this same pattern was seen, although there was a different peak value (7177 ± 654 RLU). These peaks were followed by a steady decline to 70 and 1867 RLU, respectively, after a 3 h incubation (Figure 2a,b). 

It is clear from examining a range of inducer concentrations (0.1–10 mM of *o*-CT) that the peak of bioluminescent activity occurs when 1 mM is used. The peak bioluminescence was achieved after 2 h of incubation, yet the responses were not identical using the two strains. RB1401 produced a maximum of 8783 ± 638 RLU, while KG1206 produced 3617 ± 333 RLU (Figure 2). After maximum bioluminescence activity, the decrease of bioluminescence activity was observed for all exposures and for both strains. 

**Figure 3:**
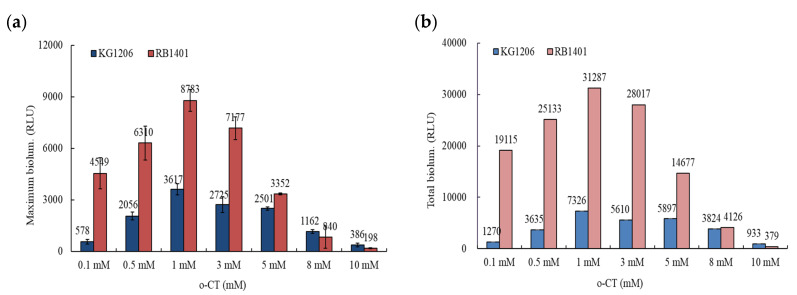
Comparisons of the bioluminescence activity between KG1206 and RB1401 exposed to various concentrations of *o*-CT: (**a**) maximum bioluminescence at each exposed concentration; (**b**) total bioluminescence production during 5 h incubation periods.

Since *o*-CT is a direct inducer for RB1401, it was expected that RB1401 would produce greater bioluminescence than KG1206. Depending on the initial inducer concentration, the difference in peak bioluminescence varied greatly. For example, at 1 mM inducer the peak for RB1401 was 2.4 times greater than for KB1206, while the same comparison at 8 mM is 0.72 (Figure 3a). This is also true when the total bioluminescence production is calculated (Figure 3b). The RB1401 strain produced in the range of 4126–31,287 RLU, which was approximately 1.1 to 15 times greater than the bioluminescence compared to that of KG1206, producing 3824–7326 RLU, except at the highly inhibited concentration of 10 mM (Figure 3b). Although the transcriptional components of the regulatory system of the *xyl*-gene are well described, its architecture is still not clear [25]. Silva-Rocha et al. [25] reported that the action of a metabolic amplifier motif (MAM) is involved in the total regulatory system of the TOL plasmid. MAM appears to express the simultaneous induction of the upper and lower (*meta*) fragments of the catabolic pathway, which would be difficult to bring about with a standard substrate responsive single promoter. However, based on the results of these preliminary experiments, we selected 1 mM of *o*-CT as the subsequent experimental conditions.

### 3.2. Effects of NPs on the Response of P_m_-lux Gene Fusion Strain, KG1206 

Following the preliminary investigations, various concentrations of individual NP were chosen to investigate the inhibition effects of NPs (ZnO, CuO, NiO, Al_2_O_3_, and TiO_2_) on the bioluminescence production of KG1206 strain with 1 mM of *o*-CT inducer. The tested concentration ranges for each NP were as follows: CuO 0–40 mg/L, ZnO 0–1 mg/L, NiO 0–2 mg/L, Al_2_O_3_, and TiO_2_ 0–5 mg/L (Table S1). The maximum bioluminescence of the control (no NP exposure) during incubation appeared in the range of 1070–2707 RLU after 2 h of incubation. 

**Figure 4:**
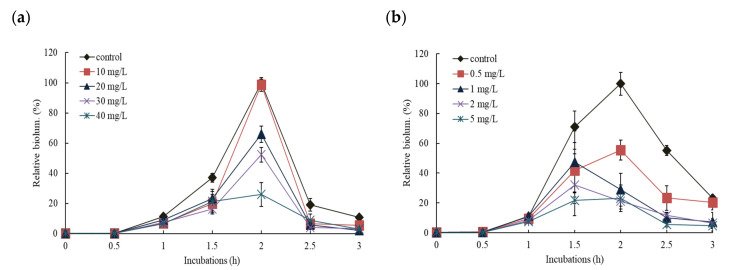
Effects of CuO and Al_2_O_3_ NPs on the bioluminescence activity of KG1206 during 3 h incubation period, expressed by the relative activity of the maximum bioluminescence of the control: (**a**) 0–40 mg/L CuO exposure; (**b**) 0–3 mg/L Al_2_O_3_ exposure.

**Figure 5:**
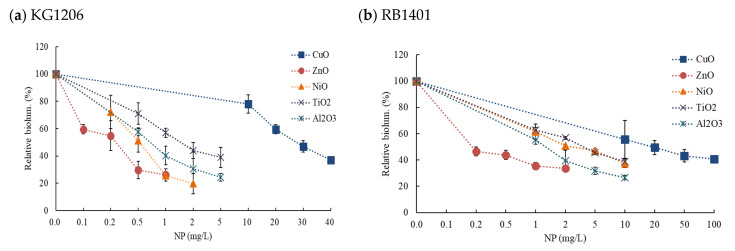
Comparisons of the changes of relative total bioluminescence with five individual NP (1 mM *o*-CT inducer): (**a**) KG1206; (**b**) RB1401 strains.

Effect patterns were slightly different depending on the exposed concentration and NP types, but maximum activity was typically observed after 2 h incubation and the activity lasted for a total of 3 h. Representative of these two NP results are shown in Figure 4. As would be expected, the higher the concentration of NP, the more inhibition of bioluminescence was seen. No stimulation of bioluminescence production was observed under tested concentrations in all cases. For the comparisons of the effects of NPs, all results were presented as the relative values (%) of total bioluminescence produced during 3 h incubation at various concentration ranges of NPs (Figure 5). Total bioluminescence is the sum of bioluminescence measured every 0.5 h from 0.5 h to 3 h of incubation periods. In these representative results, the highest and lowest exposure of each NP showed following activity levels at 2 h incubation: 99% (2056 ± 98 RLU; 1% inhibition) at 10 mg/L CuO, 3.5% (539 ± 166 RLU; 74.1% inhibition) at 40 mg/L CuO, 42% (505 ± 83 RLU; 58% inhibition) at 0.2 mg/L Al_2_O_3_, and 28% (278 ± 108 RLU; 72% inhibition) at 5 mg/L Al_2_O_3_ (Figure 4). In the case of high concentration Al_2_O_3_ NP exposure (>1 mg/L), maximum bioluminescence appeared at 1.5 h, slightly earlier than the 2 h of most conditions. Among the NPs tested, ZnO NP showed the highest bioluminescence inhibition effects, showing only 26 ± 4.5% of peak activity at 1 mg/L ZnO (max. exposed concentration). In contrast CuO NP had the lowest inhibition, showing 31 ± 9.7% relative activity at 40 mg/L CuO (maximum exposed concentration) (Figure 5a). 

### 3.3. Effects of NPs on the Response of P_u_-lux Gene Fusion Strain, RB1401 

**Figure 6:**
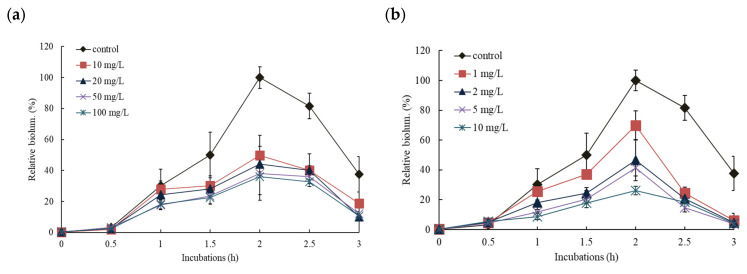
Effects of CuO and Al_2_O_3_ NPs on the bioluminescence activity of RB1401 during 3 h incubation period, expressed by the relative activity of the maximum bioluminescence of the control: (**a**) 0–100 mg/L CuO exposure; (**b**) 0–10 mg/L Al_2_O_3_ exposure.

The impact of the addition of individual NPs (ZnO, CuO, NiO, Al_2_O_3_, and TiO_2_) on the bioluminescence of RB1401 was also investigated with inducer 1 mM *o*-CT. The tested concentration ranges for each NPs were slightly different from the set used for KG1206 (CuO 0–100 mg/L, ZnO 0–2 mg/L, NiO 0–3 mg/L, Al_2_O_3_, and TiO_2_ 0–10 mg/L) although the procedure remained the same (Table S1). Representative results for two NPs (CuO and Al_2_O_3_) are shown in Figure 6, which demonstrate the effect of NP over incubation time. At the highest exposed concentration of 10 mg/L Al_2_O_3_ and 100 mg/L CuO, the maximum bioluminescence activity appeared at 26% and 36% of the control after 3 h incubation, respectively. Results are presented as the percentage of total possible bioluminescence for that strain at 3 h incubation (Figure 5b). No stimulation of bioluminescence was seen with RB1401. However, when compared to the KG1206 results, a slightly different order of the bioluminescence inhibition of exposed NPs appeared in the following order: ZnO > Al_2_O_3_ > NiO > TiO_2_ > CuO. The highest inhibition of bioluminescence production was observed at 2 mg/L ZnO (maximum exposed concentration)*,* showing relative activity 39 ± 1.7%, while the lowest inhibition at 100 mg/L CuO (maximum exposed concentration), showing relative activity 41 ± 2.7% (Figure 5b). 

### 3.4. Comparisons of the Effects of NPs on the Response of P_u_- and P_m_-lux Gene Fusion Strains

The inhibition differences of individual NPs on the bioluminescence processes of two different recombinant strains were compared using EC_50_ values, calculated based on the total bioluminescence produced during the exposure period. Of the EC_50_ values, the inhibition of NPs ranged from 0.25 mg/L (ZnO) to 26.8 mg/L (CuO) for strain KG1206, while the values ranged from 0.42 mg/L (ZnO) to 46.4 mg/L (CuO) for strain RB1401 (Table 1). The toxicity order of the NPs on the bioluminescence activity of KG1206 and RB1401 is nearly identical and was as follows: ZnO (0.25 mg/L) > NiO (0.47 mg/L), Al_2_O_3_ (0.68 mg/L) > TiO_2_ (1.57 mg/L) > CuO (26.8 mg/L) for KG1206, and ZnO (0.42 mg/L) > Al_2_O_3_ (1.58 mg/L), NiO (2.92 mg/L) > TiO_2_ (3.60 mg/L) > CuO (46.4 mg/L) for RB1401. ZnO NP caused the greatest inhibition of bioluminescence activity in both strains, while CuO had the highest EC_50s_ (i.e., the lowest bioluminescence inhibition) for both strains, being in the range of 0.47 mg/L to 1.57 mg/L and 0.42 mg/L to 3.60 mg/L for strain KG1206 and RB1401, respectively. Regardless of the type of NP, the inhibition effects on bioluminescence activity of strain KG1206 were relatively more sensitive than that of RB1401. For both strains, the difference between the lowest (ZnO) and highest (CuO) EC_50_ was approximately 100-fold. However, these results also suggest that the *P_m_-lux* gene expression could be more sensitive than that of *P_u_-lux* to NPs since the EC_50s_ value was lower than for the RB1401 strain. Though the inhibition orders of NPs on bioluminescence activity varied slightly depending on recombinant strains, the inhibition ranked in the order of ZnO > NiO, Al_2_O_3_ > TiO_2_ > CuO for both strains. 

The effect of NPs on the activity of various organisms has been extensively investigated in our laboratory, indicating that the toxicity rankings and sensitivities may depend on the organism adopted [26,27,28] (Table 2). In our previous investigation, most of the organisms tested for NP exposure showed very high EC_50s_ values for TiO_2_ NPs (>1000 mg/L, 530 mg/L; corresponding to less toxic), but in this study we found very low EC_50_ values (corresponding to high toxicity) for KG1206 (1.57 mg/L) and RB1401 (3.60 mg/L) (Table 2). 

Another bioluminescence-producing strain of *E. coli* previously investigated by this laboratory showed very different toxic effects compared to bioluminescence strains KG1206 and RB1401 used here. EC_50s_ values for bioluminescence producing *E. coli* were ZnO 1.05 mg/L, CuO 54 mg/L, NiO 198 mg/L, and TiO_2_ > 1000 mg/L, while the two strains examined in this investigation were ZnO 0.25 and 0.42 mg/L, CuO 26.8 and 46.4 mg/L, NiO 0.47 and 2.92 mg/L, and TiO_2_ 1.57 and 4.69 mg/L for KG206 and RB1401, respectively, showing approximately from 5 (ZnO) to over 700 (TiO_2_) times greater toxicity to bioreporter strains [26] (Table 2). In particular, very significant toxic differences between different bioluminescence-producing strains were observed for TiO_2_ and NiO NP exposure (Table 2). More detailed research based on the molecular level is needed to explain the reasons for these significant toxic differences. In this report, the KG1206 strain was found to be more sensitive than RB1401 for all NPs. Other researchers also reported the different effect NPs have with respect to tested organisms. For example, Lin and Xing [29] reported that ZnO NPs tested for seed germination (EC_50_ range 20–50 mg/L) was less sensitive compared to *Daphnia* (EC_50_ range 0.89–1.02 mg/L), which showed very similar sensitivity of the effects on this test [30]. All these results suggested that the appropriate assessment of NPs should be made based on test results of various organisms. 

Although the precise toxic mechanisms of NPs on the bioluminescence process are not clear at this point, many studies suggested that NP toxicity on biological systems can be affected by many factors such as solubilized metals, direct contact, characteristics of NPs (i.e., types, shapes, particle size, surface chemistry, residual impurities, etc.) as well as different potential interactions with enzymes involved on specific metabolic processes, and environmental factors [11,30,31,32,33,34,35,36,37,38]. Factors causing the negative effects might include accumulated reactive oxygen species (ROS) in bacterial systems, resulting in damage of membrane and DNA, as well as surface oxidation [39,40,41,42,43]. Studies reported that the production of ROS was induced with ZnO NPs exposure, causing membrane damage and holes in the membrane, leading to cell death by increased membrane permeability [44,45,46]. Researchers also reported that bacterial enzymes involved in metabolic processes provide numerous sites for NP adsorption and decrease potential interactions with enzymes, generating the significant negative effects on the biosynthetic and catabolic enzymatic activity [35,47]. Therefore, the binding affinities of NPs to various proteins and enzymes produced in the *lux*-gene metabolic processes can cause differences in toxic effects depending on the type of NPs and recombinant genes of *P_m_*- and *P_u_*-lux. 

In this investigation, metal concentrations in solution were determined for strain KG1206 experiments to measure the contribution of soluble metals on bioluminescence activity. Dissolved metals were observed as less than 23 µg/L Zn, 77 µg/L Ni, and 648 µg/L Cu, which correspond to 2.3%, 3.8%, and 1.6% of initially amended NPs concentration, respectively. This is a very low concentration. Therefore, the contributions of soluble metals in solution on bioreporter bioluminescence activity were thought to be insignificant or minimal in this investigation. Similar results have been reported, indicating very low solubilized metal ions and no significant concentrations for the total toxicity [48,49,50]. Bacterial systems are largely protected against NP entry. The particles themselves by the intimate contact between bacteria and NPs could be the main influencing factors on the inhibition effects of NPs, rather than the solubilized metal ions [33,51]. However, once inside the cells, the NPs act as an ion reservoir and were able to dissolve more efficiently, causing the toxicity by increasing ROS production, the oxidization of proteins, and the oxidative DNA damage. However, these could vary depending on the cellular types, biological systems, and test conditions (dose, exposure time, etc.) [50]. Some researchers suggested that complex of NPs and proteins may bind to the cell surface, which enhances cellular uptake and triggers intracellular signaling pathways, or the effect of protein-corona could reduce the interactions with cells or stabilize NPs against solubilization [52,53,54]. Some possible contaminants of synthesized NPs (e.g., synthesis, breakdown, and surface functionalization) and different conditions of culture and growth may also influence toxicity of NPs regardless of their nano-sized dimensions [35,55,56]. Therefore, the relative contribution of particles and soluble metal ions of metal-based NPs has not yet been clearly described at present and these phenomena may also involve unexpected contributions depending on the test conditions [57,58]. Consequently, focusing an understanding of the interactions between NPs and cellular or molecular mechanism of bioluminescence activity is required for future investigation. 

## 4. Conclusions

The inhibition effects of individual NPs on the bioluminescence activities of *P_m_-lux* (KG1206) and *P_u_-lux* (RB1401) bioreporter fusion strains were compared. The exposure of single NPs showed different inhibition effects depending on the NPs and recombinant strains. In particular, the activity of KG1206 was highly sensitive to the exposure of the NPs compared to the activity of RB1401. Among the NPs tested, ZnO produced the greatest inhibition effect on both strains. These inhibition phenomena can show various results depending on the laboratory test conditions. More practically, environmental contamination by NPs generally exists in mixture state and can also react with soil matrix. Other constituents can also modify their mobility, bioavailability, eco-toxicity, and other properties. Therefore, various methods should be used to examine the real effects of NPs under different conditions in future studies. Although our results showed the wide range of toxic effects of NPs that were dependent on the type of NPs and the endpoints of the tested bioluminescence systems, more systematic and molecular level research is required to clear the long-term and real toxic effects of NPs in environments. Findings in this study provide of great information to build a comprehensive understanding of the potential environmental impacts of NPs. Future studies need to investigate the effects of NPs on bioluminescent producing processes at the molecular level, and these results will provide clearly additional information about responses on NPs exposure [59].

## Figures and Tables

**Table 1:** Summary of the effects of NPs on the bioluminescence activity of two differently recombinated strains.

Strains	EC_50_ (mg/L)				
	ZnO NP	CuO NP	NiO NP	Al_2_O_3_ NP	TiO_2_ NP
KG1206	0.25 ^a^ (0.15–0.34) ^b^	26.8 (22.16–32.47)	0.47 (0.38–0.59)	0.68 (0.52–0.89)	1.57 (0.97–2.52)
RB1401	0.42 (0.11–1.54)	46.4 (14.35–80.12)	2.92 (1.76–4.84)	1.58 (1.12–2.22)	3.60 (2.12–6.12)

^a^ values represent mean of triplicates; ^b^ values in parentheses represent a 95% confidence level.

**Table 2:** Summary of previously published results of the effects of NPs on various biological activities in this laboratory.

Methods		EC_50_ (mg/L)				Reference
		ZnO	CuO	NiO	TiO_2_	
Bioluminescence activity		1.05 ^a^	54	198	>1000	[26]
Seed germination	*Lactuca*	11	0.46	17	>1000	
	*Raphanus*	46	26	114	>1000	
ATP contents		11	55	87	530	[27]
*Lactuca* root/shoot growth		0.50/>2	0.25/>1	0.57/>5	ND	[28]

^a^ values represent mean of triplicate; ND, not determined.

## Data Availability

Data can be available upon request from the authors.

## Supplementary Materials

The following are available online at https://www.mdpi.com/article/10.3390/nano11112822/s1, Table S1: Concentration ranges of tested NPs for the study of effects on the bioluminescence activity on two recombinant strains.
