# Supplementary material for: Exposure of Metal Oxide Nanoparticles on the Bioluminescence Process of Pu- and Pm-lux Recombinant P. putida mt-2 Strains
Source: Nanomaterials (Basel). 2021 Oct 24;11(11):2822. doi: 10.3390/nano11112822 (PMC8625787; doi:10.3390/nano11112822)
Supplement: Supplementary file 1 [file nanomaterials-11-02822-s001.zip › nanomaterials-1385866-supplementary.pdf]

nanomaterials

Supplementary Materials

Exposure of Metal Oxide Nanoparticles on the Bioluminescence Process of P_u- and P_m-*lux* Recombinant *P. putida mt-2* Strains

In Chul Kong [1], Kyung-Seok Ko [2,*], Sohyeon Lee [1], Dong-Chan Koh [2] and R. S. Burlage [3]

1 Department of Environmental Engineering, Yeungnam University, Gyungsan 38541, Korea; ickong@ynu.ac.kr (I.C.K.); lswsh0803@naver.com (S.L.)
2 Groundwater Department, Geologic Environment Division, Korea Institute of Geoscience and Mineral Resources (KIGAM), Daejeon 34132, Korea; chankoh@kigam.re.kr
3 Department of Pharmaceutical and Administrative Sciences, Concordia University, Mequon, WI, USA; Robert.burlage@cuw.edu
* Correspondence: kyungsok@kigam.re.kr; Tel.: +82-42-868-3162

Table S1. Concentration ranges of tested NPs for the study of effects on the bioluminescence activity on two recombinant strains.

NPs (mg/L)	Strains	
	KG1206	RB1406
ZnO	0.1, 0.2, 0.5, 1	0.2, 0.5, 1, 2
CuO	10, 20, 30, 40	10, 20, 50, 100
NiO	0.2, 0.5, 1, 2	1, 2, 5, 10
TiO_2	1, 2, 5, 10	1, 2, 5, 10
Al_2O_3	0.5, 1, 2, 5	1, 2, 5, 10
